# Analyse des risques perçus des prestataires de santé en milieu hospitalier dans le cadre de la pandémie à COVID-19: une étude qualitative dans le Centre Hospitalier Roi Baudoin de Guédiawaye, lors de la réception du 1^er^ cas communautaire du Sénégal

**DOI:** 10.11604/pamj.supp.2020.37.1.25389

**Published:** 2020-10-13

**Authors:** Ndèye Marème Sougou, Jean Baptiste Diouf, Amadou Amath Diallo, Ibrahima Seck

**Affiliations:** 1 Département de Médecine Préventive et Santé Publique, Université Cheikh Anta Diop, Dakar, Sénégal,; 2 Institut de Santé et Développement, Université Cheikh Anta Diop, Dakar, Sénégal,; 3 UMI3189 « Environnement, Santé, Sociétés », UCAD, CNRS, CNRST, USTTB, UGB, Saint-Louis, Sénégal,; 4 Hôpital Roi Baudoin, Guédiawaye, Sénégal

**Keywords:** Risques perçus/réels, personnels de santé, Sénégal, Perceived/actual risks, healthcare personnel, Senegal

## Abstract

**Introduction:**

les systèmes de soins en Afrique de l´Ouest ont été mis à rude épreuve depuis le début de la pandémie à COVID-19. L'exposition du personnel de santé à l'infection dans le cadre de l´épidémie à la COVID-19 a été évoquée dans plusieurs études. L´objectif de cette étude est d´analyser les risques perçus et réels par le personnel de santé dans le premier hôpital à avoir reçu un cas communautaire de COVID-19 au Sénégal.

**Méthodes:**

une étude descriptive exploratrice de la perception des prestataires de santé vis à vis du risque encouru lors de la COVID-19 avait été fait. Quarante-sept prestataires de santé ont fait l´objet d´entretien individuel approfondis dans cet hôpital.

**Résultats:**

la crainte de la maladie était bien présente auprès du personnel de santé. Cette crainte était la résultante de plusieurs facteurs exogènes et endogènes parmi lesquels on comptait la méconnaissance du virus et de la maladie à COVID-19, le sentiment de vulnérabilité liée à une insuffisance dans la disponibilité des matériels de protection individuels, la position du prestataire par rapport au sacerdoce de la profession médicale et le risque réel/ perçu d´être un potentiel danger pour leur famille et leur entourage.

**Conclusion:**

cette étude a pu faire ressortir la nécessité de la prise en charge psycho-affective des personnels de santé dans le cadre de cette pandémie en prenant en compte la dimension genre. Une mise à disposition suffisante d´équipement de protection individuelle et de mesures de gestion du stress pourraient permettre aux personnels en première ligne de faire face à cette pandémie en toute sérénité.

## Abstract (en)

**Introduction:**

health care systems in West Africa have been under strain since the beginning of the COVID-19 pandemic. The exposure of health personnel to infection during the COVID-19 pandemic has been reported in several studies. The purpose of this study was to analyze health workers’ actual and perceived risk in the first hospital faced with managing a community-acquired COVID-19 case in Senegal.

**Methods:**

we conducted an exploratory descriptive study of health care providers’ perception about their own risk during the COVID-19 pandemic. Forty-seven health-care providers were interviewed in personal, extensive interviews in this hospital.

**Results:**

the fear of disease was present among the health staff. This fear was caused by several exogenous and endogenous factors, including the lack of knowledge of the virus and COVID-19 disease, the feeling of vulnerability due to insufficient availability of personal protective equipment, healthcare professionals’ position in relation to the priesthood from the medical profession and the real and perceived risk of putting their family and their entourage in potentially dangerous situations.

**Conclusion:**

this study highlights the need for psycho-affective management of health care workers during this pandemic by taking the gender dimension into account. The provision of personal adequate protective equipment and stress management measures could enable front-line workers to cope with this pandemic in complete serenity.

## Introduction

L'épidémie actuelle de coronavirus (COVID-19) est dévastatrice, malgré la mise en œuvre de mesures de contrôle à grande échelle. L'épidémie a débuté à Wuhan, la capitale de la province du Hubei en Chine, et s'est rapidement étendue aux différentes régions du Hubei et à toutes les autres provinces chinoises. Rapidement cette dernière s´est propagée dans les différents continents du monde. Le continent africain est l'un des derniers à être touché après l'Europe et les États Unis. L'Afrique a enregistré son premier cas officiellement le 15 février 2020, deux mois après qu´il soit identifié pour la première fois en Chine [1]. L´évolution de la COVID-19 au Sénégal, à l´instar des autres pays d´Afrique de l´Ouest a suivi une évolution croissante atteignant des milliers de cas depuis l´introduction de son premier cas à la date du 3 Mars 2020 [2,3].

La COVID-19 devient dès lors une crise immédiate se comportant comme un agent pathogène qui ne se produit qu'une fois par siècle et qui crée des inquiétudes surtout dans les pays africains et de l´Asie du Sud [4]. Cette inquiétude est d´autant plus importante que dans ces pays en développement, les systèmes de santé ne sont pas assez préparés pour faire face à des crises sanitaires en atteste la réponse lente et insuffisante des pays d´Afrique de l´Ouest lors de l'épidémie à Ebola en 2014 [5]. Les scenarii décrits par la littérature scientifique prédisent pour les 46 pays d'Afrique subsaharienne, qui abritent plus d'un milliard de personnes, une trajectoire de l´épidémie de la COVID-19 similaire à celle de la grippe aviaire hautement pathogène dans le nord du monde [6]. Des études ont également montré que la charge létale de l´infection à COVID-19 était proportionnelle à la résilience des systèmes de soins [7]. Les systèmes de soins sont composés des ressources humaines en santé. L'exposition du personnel de santé à l'infection dans le cadre de l´épidémie à la COVID-19 a été évoquée dans plusieurs études. En effet, en Chine, on estime que 3000 travailleurs de la santé ont été infectés et qu'au moins 22 sont morts. Dans une recherche des descripteurs vulnérabilité, risque, et risques pour la santé professionnelle et personnelle, établissant un échantillon de 21 articles, les risques et les vulnérabilités du personnel de santé étaient liés en partie au manque de ressources nécessaires (exemple) pour le travail, à la violence physique et à la tension émotionnelle [8]. Peu d´études ont été retrouvé concernant l´impact de l´épidémie sur les ressources humaines en santé dans les hôpitaux en Afrique de l´Ouest. La perception du risque a été théorisée comme étant intiment liée aux émotions et la prise de décision [9].

L´une des fonctions des émotions liées à la prise de risque est de permettre des choix rapides sous la pression du temps mais aussi de générer des engagements concernant des décisions moralement et socialement significatives [10]. L´intérêt de cette étude de la perception du risque chez les prestataires de santé permettrait de fournir une base pour comprendre et anticiper les réactions des professionnels de santé et d´améliorer la communication des informations sur les risques liés à la COVID-19 en milieu professionnel sanitaire. L´objectif de cette étude est d´analyser les perceptions du risque professionnel et de la vulnérabilité dans le cadre de l´épidémie de la COVID-19 dans le premier hôpital à avoir reçu un cas communautaire de COVID-19 au Sénégal. Cette étude permettra d´élaborer et de mettre en œuvre des politiques publiques en matière de santé des travailleurs afin d'améliorer les conditions de travail dans le cadre de la COVID-19 et d´apporter une réponse adaptée et contextualisée des besoins des professionnels de santé dans le cadre de la réponse au COVID-19.

## Méthodes

**Cadre d´étude:** le Centre Hospitalier Roi Baudouin (CHRB) est situé dans le département de Guédiawaye qui est l´un des quatre départements que compte la région de Dakar. Ce département est situé dans la zone périurbaine de Dakar avec une population estimée à 360 360 habitants soit une densité de 13 447 habitants au Km^2^ contre 66 habitants au Km^2^ pour l´ensemble du pays et 6 409 Habitants Km^2^ pour la région de Dakar. L´hôpital Roi Baudoin de Guédiawaye a reçu le 1^er^ cas communautaire de COVID-19 au Sénégal le 02 mars 2020.

**Type d´étude:** il s´agissait d´une étude descriptive exploratrice de la perception des prestataires de santé vis à vis du risque encouru lors de la COVID-19.

**Population d´étude:** la population d´étude est composée des prestataires de santé, hommes et femmes qui travaillent à Centre hospitalier Roi Baudoin de Guédiawaye. Les différents types de fonctions seront pris en compte (médical, paramédical, personnel administratif, personnel de soutien). Étaient inclus dans l´étude le personnel de santé travaillant à l´hôpital de Roi Baudoin depuis au moins 1 an, celui n´étant pas en fonction au moment du contact avec le premier patient dépisté COVID+ au niveau de l´hôpital et celui acceptant de participer à l´étude.

**Collecte des données:** cette étude s´était déroulée du 1er Juin au 30 juin 2020 et a duré 30 jours pour la collecte des données. Des entretiens individuels approfondis encore appelés interviews avaient été menés. Durant cette période, les interviews ont été menés en face-to-face dans des lieux privés à la discrétion des participants et permettant de respecter les règles de distanciation sociale. L'enquête a été menée par des chercheurs spécialisés en anthropologie et ayant une expérience professionnelle dans le domaine de la santé. Les conversations duraient généralement une heure. Un guide d'interview avec des questions ouvertes avait été utilisé par les enquêteurs durant tous les interviews [11]. Les principales questions abordées concernaient les connaissances et appréciations concernant la pandémie à COVID-19, la perception du risque encouru, les expériences des professionnels vis à vis des situations de risque en milieu hospitalier et des particularités de genre. Toutes les interviews avaient été enregistrées sur bande magnétique.

**Analyse des données:** les données qualitatives avaient été transcrites mot pour mot selon des règles de transcription modifiées par Kallmeyer et Schütze [12]. Un échantillon aléatoire de transcriptions et de traductions avait été soumis à un contrôle de qualité par l'écoute d'enregistrements audio et de contre-traductions et des mesures avaient été prises en conséquence. Après s'être familiarisé avec tous les entretiens/documents de synthèse, un codage des transcriptions avait été fait. Les données retranscrites étaient analysées en utilisant la méthode de l´analyse thématique. À cet effet, l´analyse consistait, dans un premier temps, à extraire des transcriptions les fragments ou blocs de sens les plus représentatifs de la position de la personne (analyse verticale) et, dans un deuxième temps, à classer ces extraits par catégories (analyse horizontale). Le logiciel d´analyse qualitative Nvivo 12 avait été utilisé.

## Résultats

Les données des résultats ainsi présentées avaient été collectées auprès de 47 enquêtés qui tous travaillent au centre hospitalier Roi Baudoin. Le nombre de prestataires enquêtés étaient de 47. Il s´agissait de 33 femmes et de 14 hommes. À travers une démarche de diversification externe [13], nous nous sommes entretenus avec 25 personnels paramédicaux, 13 médicaux, 2 membres du personnel administratif et de 7 personnels de soutien.

**Figure 1:**
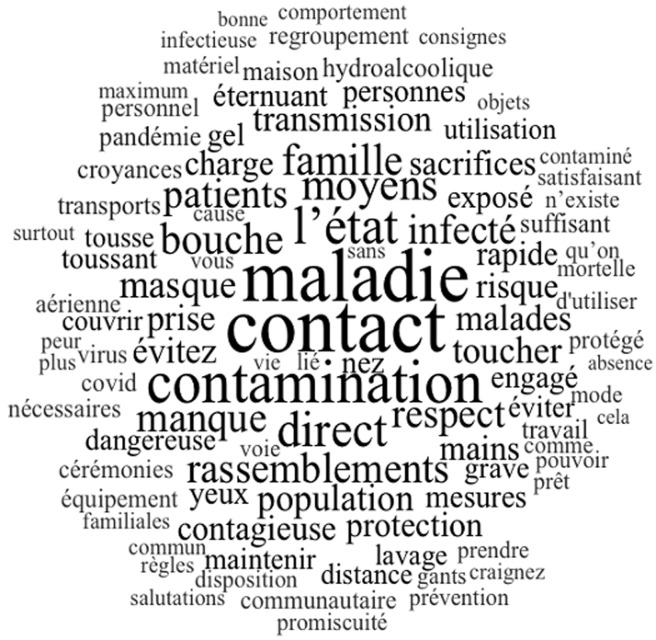
nuage de mots -discours des prestataires

**Perception de la maladie à COVID-19 par les prestataires:** la maladie à COVID-19 est considérée comme une maladie grave et surtout contagieuse par les prestataires «*La maladie est une pandémie à déclaration obligatoire, très contagieuse pouvant engager le pronostic vital de la personne contaminée*» (personnel médical). La contagiosité est une des préoccupations majeures des prestataires enquêtés (Figure 1). Pour la plupart de ces derniers, la transmission de la maladie est rapide et est souvent lié à un contact direct. Dans leur situation, il s´agira probablement d´un contact avec les patients. Sa dangerosité et gravité sont liées au fait que c´est une maladie mortelle selon eux. Nouvelle selon eux, cette maladie n´a pas encore de traitement d´où l´intérêt d´être bien protégé afin de s´en prémunir en atteste les propos de cette femme médecin de 44 ans: «*La COVID-19 est une maladie nouvelle, donc mal connue et de ce fait elle est dangereuse..*.» Le mot maladie, contact et contamination reviennent souvent dans le discours des patients. La contamination est la principale préoccupation des prestataires de santé (Figure 1).

Selon les agents médicaux, les causes de la transmission «*rapide*» de la pandémie à COVID sont nombreuses. Pour eux, on retrouve au niveau de la communauté, une méconnaissance de la maladie. Cette méconnaissance de la maladie est en partie liée au fait qu´il y a un niveau d´éducation faible au sein de la société. Il existe dès lors dans cette communauté des comportements en faveur de la rapidité de la transmission de la maladie. On dénombre parmi ces comportements, une propension élevée à se rassembler avec l´organisation de cérémonies familiales et l´existence de déplacements interrégionaux comme l´affirme AD, Médecin, 29 ans, homme: «*il existe un non-respect des mesures mises en place par l´état ; par exemple il y a une mobilité des personnes. Malheureusement ces personnes constituent les vecteurs du virus*». La plupart des gens qui vivent à Dakar ont leur famille dans les régions intérieures. De plus, on note l´existence de grandes familles dans les sociétés sénégalais comme l´avance cette femme, paramédical 51 ans Paramédical, femme 51 ans: «*Vous savez, au Sénégal, les populations vivent dans de grandes familles, cela rend très difficiles le respect des mesures barrières dans le cadre de la COVID-19*». La promiscuité est la règle dans la plupart des familles. Selon les prestataires de santé, ces conditions sociales n´aident pas au respect des conditions d´hygiènes et des mesures de prévention édictées par les autorités dans la cadre de la lutte contre le COVID-19.

Seul le respect des mesures de prévention pourrait permettre aux communautés d´être protégés de l´infection à COVID et de limiter la transmission du virus. Selon eux il existe des rumeurs sur des supposés facteurs protecteurs en Afrique. Il s´agirait selon eux de l´existence de la chaleur ou encore du type négroïde des africains. Ces rumeurs seraient infondées et liées à des croyances et croyances culturelles sur la maladie à COVID-19. Paramédical, homme, 50 ans: «*les facteurs protecteurs, (oh), ce sont des croyances simplement. Dès le début c´est ce qu´on croyait. On avait pensé que l´africain avec son taux de mélanine et les températures chaudes ne constituait un danger face à la maladie. Mais que nenni, certes il y a pas encore de mort au Sénégal mais le nombre ne cesse de croitre*». Les données révèlent que chez les prestataires les perceptions et les savoirs sur le virus sont construits sur une bonne dose de recul critique tant face aux discours experts que face à ceux véhiculés par les médias de masse. Il n'a pas été noté une référence à la création du virus ni à une à quelconque “théorie du complot”.

**Crainte de l´infection/ sentiment de vulnérabilité à l'infection:** il ressort de l'analyse des données que les prestataires sont animés par un sentiment de vulnérabilité à l'infection. En effet, ce sentiment de vulnérabilité se rapporte selon eux aux faits qu'ils sont en contacts direct avec des patients déclarés positifs pour ceux qui sont déjà testés et d'autres qui viennent pour se faire tester. Alors la peur et l'angoisse sont les sentiments les mieux partagés par les prestataires quel que soit leur position dans le système de soin. La peur est associée à la crainte d´être infecté aussi bien dans le (personnel soignant que le personnel de soutien). Les propos rapporté par BF, 33 ans médecin: «*J´ai peur d´être infecté parce que les moyens manquent dans notre structure, ainsi nous ne sommes pas assez protégés en tant que personnel médical*». D'ailleurs, ces prestataires se considèrent plus vulnérables à l'infection que le reste de la population en raison de leur position professionnelle et ils sont sans rempart. Les raisons évoquées qui accentuent ce sentiment de vulnérabilité chez les prestataires vont au-delà de leur position professionnelle et touchent à leur sous-équipement en matériel de protection. Ces derniers soulignent par ailleurs que les raisons de leur crainte seraient liés au fait qu´ils ne soient pas assez équipés en matériel de protection par leur structure de santé pour faire face à cette maladie. L´ ampleur de la crainte que leur inspire le virus est grande et peut être comparé à des forces démoniaques d´après certains propos. C´est de cas de OD, 22 ans personnel de soutien qui affirme que «*le virus est démoniaque, nous sommes tous exposés*».

**Perception de la protection individuelle des prestataires:** la majorité des prestataires ne se sentent pas bien protégées dans l´exercice de leur fonction. Les équipements (masques, gants) mis à disposition par l´hôpital leur semble insuffisant. Certains affirment prendre des dispositions particulières personnelles pour se protéger. Il s´agit surtout de l´achat de masque. Cette situation les place quelque fois en inconfort face à des collègues non protégés car n´ayant pas les moyens d´acheter leur propre masque. Personnel paramédical, 27 ans, Femme, «*non, nous ne sommes pas bien protégée car il n´y a pas assez de provisions en gants et masques. De plus, il n´y a aucune barrière entre nous et les malades*».

**Les prestataires entre exigences éthiques, risque encouru et sentiment de responsabilité extrahospitalière:** selon les prestataires Les risques encourus sont de 2 types. Le risque encouru est d´abord individuel. Il est en rapport avec le risque pris par le prestataire lui-même dans l´exercice de ces fonctions. D´autre part le risque est corrélé au risque encouru par la famille et l´environnement social du prestataire. Dans ce dernier cas, le prestataire devient un risque pour son entourage. Les risques entourant l'infection et la contamination des proches semblent encore accentuer le sentiment de vulnérabilité. Le prestataire encoure dès lors le risque d´être stigmatisé par sa propre famille. C´est le cas de jeune femme de 32 ans exerçant une profession paramédicale et qui est stigmatisée par sa famille «*Ma famille a peur et dès que je rentre du travail, personne ne s´approche de moi, c´est malheureux bien que je sois consciente que c´est moi qui ai opté de faire la santé*» De plus, les familles de ces prestataires sont à leur tour stigmatisés par leur voisinage qui les fuient. Toutefois, l´ambiance de panique a exacerbé le sentiment de responsabilité envers les proches. Cet argument lié à la responsabilité envers les proches vulnérables fut décliné en plusieurs variantes dans le discours de nos participants. Le risque encouru par la famille du prestataire est réel selon certains prestataires. La perception de ce risque est d´autant plus accrue que certains prestataires affirment que les équipements de protection mis à leur disposition sont insuffisants ce qui augmente la vulnérabilité de leur famille. Ainsi ce paramédical de 50 ans : «*le risque pour ma famille est énorme et très élevé. Cela à cause du manque d´équipement ou de moyens de prévention qui a été mis à notre disposition*». Les mesures personnelles de se prémunir se rapportent plus à une logique de responsabilité (culpabilité d´avoir contaminé des proches) que d´une logique épidémiologique de lutte contre la propagation du virus.

**Niveau d´engagement consenti par le prestataire dans le cadre de la prestation de lutte contre le COVID-19:** le niveau d´engagement des prestataires pour faire face à la pandémie est assujetti à certaines conditions. Ainsi, la plupart se sentent engagés en tant que soignants à prendre en charge les cas dans la mesure où les conditions sécuritaires sont réunies. Ces conditions étaient surtout la mise à disposition d´équipement de protection en quantité suffisantes dans la structure de santé. Cette femme de 36 ans paramédical affirme juger son niveau d´engagement satisfaisant dépendant des mesures de protections mis à leur disposition: «*Moi je juge mon niveau d´engagement satisfaisant, je suis prêt pour la prise en charge à condition que nous disposons d´outils nécessaires pour être protégés de patients contaminés*». Certains prestataires au-delà de l´engagement parlent de sacrifices. Pour ces derniers, peu nombreux, les sacrifices consentis pourraient être importants. Certains se disent prêt à se sacrifier pour prendre en charge les cas. La réquisition par le gouvernement du personnel sanitaire selon eux pourrait être un scénario possible dans le cadre de la riposte à COVID-19. Dans cette éventualité, ils seraient prêts à s´y soumettre volontiers. Leurs compétences et leur temps seraient dès lors sacrifié pour «sauver les patients». L´exemple de ce médecin homme de 34 ans est illustratif: «*Personnellement, je suis prêt à donner mon temps et mes compétences pour sauver des vies dans le cadre de cette maladie*». Cependant, il a été recensé un nombre limité de prestataires ne sentant pas prêt à faire face à l´affluence de malades souffrant de COVID-19. Ces derniers disent ne consentir à aucun sacrifice pour lutter contre l´épidémie. On les dénombre dans tous les corps du personnel hospitalier (personnels de soutien, médecin, paramédicaux).

**Difficultés spécifiques liées au genre/sexe dans la prestation de service contre le COVID-19:** des difficultés relatives au sexe des prestataires ont pu être soulignées par cette étude. En effet, certaines femmes prestataires de santé signalent des difficultés particulières dans l´exercice de leur fonction. Il s´agissait surtout de jeune femme qui sont mères de jeunes enfants. Les horaires revus dans le cadre de l´épidémie avec une extension des heures de travail impactent sur la qualité de leur vie familiale surtout dans le maternage de leurs enfants. Dans cette population féminine, certaines ont des enfants en bas âge qui nécessite encore une attention particulière. On y dénombrait même des femmes enceintes et allaitantes. Toutes les catégories de personnels hospitalier étaient concernées. Ainsi on peut rapporter les dires de AS, 39 ans, personnel administratif: «*J´ai des difficultés comme toute autre femme qui travaille, on n’a pas le temps de surveiller nos enfants» ou en encore FT, 35 ans, femme médecin: «Mon implication dans cette crise épidémique a fait que je n´ai plus assez de temps pour la maison, mes enfants surtout et la gestion du quotidien familial*». De plus pour la plupart de ces femmes interrogées, à ces difficultés familiales, s´ajoute la charge émotionnelle liée à la peur de contaminer leurs propres enfants.

## Discussion

Notre étude a pu mettre en exergue l´importance de la prise en compte du risque perçu par le prestataire de santé dans le cadre de la gestion de la prise en charge des soins lors de la pandémie à COVID-19. Au Sénégal dans ce premier hôpital en contact avec le 1er cas communautaire de COVID-19, il est ressorti que la crainte de la maladie est bien présente auprès du personnel soignant. Cette crainte serait la résultante de plusieurs facteurs exogènes et endogènes. Il s´agirait de la méconnaissance du virus et de la maladie à COVID-19, du sentiment de vulnérabilité liée à une insuffisance de matériels de protection individuels, de la position du prestataire par rapport au sacerdoce de la profession médicale et du risque réel/ perçu d´être un potentiel danger pour leur famille et leur entourage. Cette situation évoque la notion de rapport au risque. Chez les prestataires rencontrés, le rapport au risque relève, non pas d´une logique individuelle, mais de la conjugaison d´une pluralité de facteurs qui déterminent une ou plusieurs formes de rationalités mises à contribution. Le sentiment de vulnérabilité implique de la part des prestataires un nouveau rapport à soi, aux autres et au monde, à son métier l'éthique voire à l´intégration du risque dans toutes les interactions. Dans le cas d'espèce, le concept de situation à risque est applicable aux prestataires et c'est ce qui exacerbe leur angoisse. Il s´agit de situation à risque d'être contaminé à l'hôpital avec les collègues, les patients et en famille du risque de contaminer les proches et les parents.

Une mauvaise connaissance du virus avait été identifiée auprès du personnel hospitalier. Les canaux d´information formels n´étaient pas encore mis en place afin de permettre une connaissance éclairée du virus. Ainsi, les prestataires enquêtés perçoivent le virus comme dangereux, mortel et d´une grande contagiosité. Ces derniers avouaient que le virus et son comportement étaient mal connus. D´autres études, ailleurs, avaient pu montrer que beaucoup de prestataires connaissaient mal le virus. En Inde, une proportion significative d'agents de santé connaissait mal sa transmission (n=276, 61,0%) et l'apparition des symptômes (n=288, 63,6%) [14]. Pourtant en Égypte, une étude avait montré qu´il existait une corrélation positive entre les scores de connaissance concernant la COVID-19 et l'attitude des prestataires [15]. Dans cette même étude faite en Egypte, la plupart des prestataires enquêtés (83,1%) déclaraient qu'ils avaient peur d'être infectés par COVID-19, et 89,2% déclaraient qu'ils étaient plus susceptibles d'être infectés par COVID-19 que les autres [15]. Ces résultats sont similaires à ceux de notre étude. En effet, les prestataires interviewés ont développé une réelle crainte de la maladie à COVID-19. Se sentant plus vulnérables que les communautés car étant en contact direct avec les patients infectés. Ce sentiment de crainte est exacerbé par un sentiment de vulnérabilité lié à une insuffisance des mesures de protection mis à leur disposition dans la structure de santé. C´est le cas dans d´autres structures dans des pays en développement. C´est ainsi qu´en Amérique latine, les travailleurs de la santé avaient également eu un accès limité aux équipements de protection individuel essentiel et au soutien des autorités sanitaires pendant la pandémie COVID-19 [16].

La situation de la pandémie à COVID-19 étant une cause potentielle d´inquiétude, a été mise en rapport avec une augmentation du niveau de stress lié au travail chez les professionnels de santé [17]. La littérature scientifique l´a également identifié comme étant une source de peur dans la communauté de professionnel de soins [18]. Cette peur des prestataires se rapporte au fait qu'ils naviguent entre des exigences contradictoires d'une part le sacerdoce lié à rôle en milieu hospitalier ; d'autre une prise de conscience du risque encouru. Ce dernier aspect relatif à leur prise de conscience du risque s'explique par leur appartenance au milieu médical et c'est-à-dire à la «culture du risque» [19]. Par ailleurs, la peur dans d´autres contextes a modifié le comportement des praticiens [18]. Loin d'être irrationnelle, elle permet aux concernés au contraire de prendre les meilleures décisions pour se prémunir dans certains cas. Il n´est donc pas étonnant qu´il y ait eu un impact sur le niveau d´engagement des prestataires dans le cadre de l´exercice de leur fonction dans cet hôpital de la banlieue dakaroise. Ainsi notre étude a pu montrer une baisse de l´engagement des prestataires dans le cadre de l´exercice de leur fonction. La majeure partie ne se sentant pas protégée et se sentant surexposée admettent qu´ils ne se sacrifieraient pas dans le cadre de la gestion de l´épidémie à COVID-19 [18,20]. Faire partie de la profession médicale implique d'assumer le fardeau du risque clinique et d'accepter implicitement le sacrifice personnel [21]. La pandémie actuelle à COVID-19 appelle à reconsidérer la notion de sacrifice dans le contexte d'un risque individuel et sociétal supplémentaire; car les conduites et comportements dits à risque ont une signification pour les individus, en lien avec des croyances, des rites, un besoin de réalisation de soi, d'affirmation identitaire et d'intégration sociale [21,22]. Pour dire dans ce sens à côté des exigences éthiques et professionnelles, les comportements et les pratiques des prestataires s´inscrivent dans une réflexivité et donc, dans une révision constante des pratiques [22]. Le risque zéro n'existant nul part, la gestion des risques n´est aucunement naïve. Toute attitude face à un risque procède d'une évaluation, celle-ci s'appuie sur valeurs propres, en adéquation avec le mode de construction du risque à l'œuvre dans la collectivité d´appartenance [9,23].

La plupart des études faites sur l´affect de la COVID-19 sur les familles et le voisinage ont concernés les familles des patients infectés par la COVID-19. Ces études ont montré une charge émotionnelle importante relative à la peur dans l´entourage des patients [24]. Notre étude a pu montrer qu´à l´instar des patients COVID-19+, les familles des prestataires de santé également se sentaient exposées à la maladie et faisaient face à un stress permanent. Ce stress est à l´origine quelquefois d´une stigmatisation des prestataires eux-mêmes au sein de leur cellule familiale dans certains cas. Des études ont montré que souvent lors d´épidémies, les personnels de santé ont été stigmatisés et ostracisés [25]. Dans cette étude, des difficultés particulières liées au sexe et au genre du prestataire de santé ont été mis en exergue. En effet certains prestataires de sexe féminin ayant un rôle de maternage de leurs enfants avaient peur d´infecter ces derniers. Par ailleurs, la charge de travail augmente avec de nouveaux horaires auraient, selon elles, des répercussions sur leur vie de famille et plus particulièrement sur leurs responsabilités de maternage et leur rôle reproductif au sein de la famille. Cette préoccupation supplémentaire aurait des répercussions sur l´anxiété causée par cette pandémie. D´autres études avaient montré que la pandémie de COVID-19 pouvait affecter de manière significative la santé mentale des travailleurs de la santé, qui se trouvaient en première ligne de cette crise. Des différences entre les sexes avaient mis en exergue par cette dernière. Ainsi, les femmes médecins et infirmières présentant des taux plus élevés de symptômes affectifs que les hommes [26].

## Conclusion

Cette étude a pu montrer l´importance des méthodes qualitatives dans la gestion des crises épidémiques. Nos résultats confirment que l´anthropologie a permis de dépasser les théorisations décontextualisées et discours savants autour du risque pour s´engager dans une analyse ethnographique à partir du risque tel qu´il se vit et est négocié au quotidien au sein de la famille et du réseau social. L´analyse montre que la construction populaire du risque épidémique est profondément influencée par un système local de valeurs, une dynamique de négociation entre les membres du réseau social restreint, d´attentes en termes de transparence et de cohésion de la part du savoir expert et d´une bonne dose de recul critique face aux « évidences scientifiques ». En milieu hospitalier, cette étude a permis d´identifier les craintes des personnels de santé face à l´épidémie à COVID-19. Elle a également permis de mettre en évidence les modifications de comportements des prestataires face au stress et à la charge émotionnelle causée par l'exposition à une potentielle contamination à la COVID-19. Il en ressort une nécessité de prise en charge psycho-affective des personnels de santé dans le cadre de cette pandémie en prenant en compte le genre. Par ailleurs, un meilleur engagement des autorités sanitaires par une mise à disposition d´équipement de protection individuelle et des mesures de gestion du stress pourraient permettre aux personnels en première ligne de faire face à cette pandémie en toute sérénité.

### Etat des connaissances sur le sujet

- En situation de pandémie, les ressources humaines sont impactées par les risques et les vulnérabilités;
- Les risques et les vulnérabilités du personnel de santé sont liés le plus souvent en partie au manque de ressources nécessaires (exemple) pour le travail, à la violence physique et à la tension émotionnelle.

### Contribution de notre étude à la connaissance

- Parmi les principaux facteurs responsables des craintes des personnels de santé en milieu hospitalier face à l´épidémie à COVID-19, est dénombrée l´insuffisance des mesures de protection individuelle au sein des structures de santé;
- La gestion psychoaffective du stress des ressources humaines en santé dans le cadre de la pandémie doit prendre en compte la dimension genre.
